# Attentional bias in tobacco use disorder using eye tracking: A systematic review

**DOI:** 10.1016/j.dadr.2024.100294

**Published:** 2024-11-02

**Authors:** Noreen Rahmani, Alma Rahimi, Kameron Iturralde, Laurie Zawertailo

**Affiliations:** a University of Toronto, Department of Pharmacology & Toxicology, Toronto, Canada; b Centre for Addiction and Mental Health, INTREPID Lab, Toronto, Canada; c University of Western Ontario, Schulich School of Medicine & Dentistry, Faculty of Neuroscience, London, Canada

**Keywords:** Attentional bias, Tobacco, Tobacco use disorder, Smoking, Cigarettes, Eye tracking

## Abstract

**Background:**

Attentional bias, defined as the disproportionate attentional allocation towards drug-related stimuli, is well-demonstrated in substance use disorders. However, studies investigating attentional bias in tobacco use disorder have revealed inconclusive findings. In recent years, eye-tracking technology has emerged as an innovative technique for exploring attentional bias. This systematic review aims to provide a comprehensive overview of eye-tracking studies examining attentional bias in tobacco use disorder.

**Methods:**

Using PRISMA guidelines, 18 papers that assessed attentional bias using eye-tracking technology among people who smoke cigarettes were extracted from the following databases: PsychINFO, MEDLINE, and EMBASE. Search terms included “attentional bias”, “tobacco use disorder”, and “eye tracking” and their respective subject headings and synonyms. Selected papers were assessed for methodological quality using a standardized procedure. Selected studies reviewed were categorized into studies making comparisons between 1) people who smoke and people who do not smoke and 2) between smoking-related cues and neutral cues among people who smoke.

**Results:**

Overall, most studies showed that people who smoke had significantly greater attentional bias to smoking-related cues, as indexed by greater dwell times and fixation counts. Although findings using measures of early orienting biases were mixed, people who smoke displayed a tendency to initially shift attention to smoking-related cues more frequently than neutral cues.

**Conclusions:**

While methodological inconsistencies across studies preclude any definitive conclusions, findings suggest that maintained attention may be a more precise reflection of the specific attentional processes influenced by incentive salience. Suggestions for future research include establishing methodological standards for future eye-tracking studies.

## Highlights

• We review eye-tracking studies examining attentional bias in tobacco use disorder.
• Studies found greater attentional bias to smoking cues in people who smoke than those who don't.
• Attentional bias relies more on maintained attention than early attentional shifts.
• Future eye-tracking studies should adopt a standardized methodology.

## 1 Introduction

Tobacco use is one of the leading causes of preventable death worldwide (Reitsma et al., 2021). Globally, smoking tobacco accounts for approximately 7.7 million deaths and 200 million disability-adjusted life years each year (Reitsma et al., 2021). With about 1.14 billion people globally who smoke tobacco, the World Health Organization considers tobacco use one of the greatest threats to public health (Reitsma et al., 2021, WHO, 2022).

Tobacco use disorder is an addiction defined by the Diagnostic and Statistical Manual of Mental Disorders, fifth edition (DSM-5) to be a problematic use of tobacco leading to clinically significant distress or impairment (APA, 2013). Within the addiction science literature, it is well established that cue associations are fundamental to the development and maintenance of addictive behaviours. The theory of incentive salience, a highly influential theoretical model of addiction, posits that repeated associative pairings with drug-related cues lead to sensitization in neurobiological pathways associated with motivation and drug salience (Robinson and Berridge, 1993, Robinson and Berridge, 2001). As a result, these drug-related cues can elicit conditioned dopaminergic responses, causing individuals to perceive these cues as highly attractive and attention-grabbing. This disproportionate attentional allocation towards incentive cues is known as attentional bias and is believed to play an important role in addiction-related behaviours such as drug-seeking, craving and recurrence of use (Field and Cox, 2008a, Franken, 2003).

Attentional bias is suggested to have a mutual excitatory relationship with substance craving, where increases in cravings lead to increases in attentional bias toward a substance-related cue and vice versa (Field and Cox, 2008a, Franken, 2003). In situations where conditioned substance-related cues are present, these two processes can positively influence one another, ultimately motivating drug-seeking behaviour and maintaining substance use (Franken, 2003). It has been proposed that this relationship also functions in the case where the substance is perceived as unavailable (i.e., when an individual is in treatment), leading to decreases in both subjective cravings and attentional bias (Field and Cox, 2008a). Therefore, substance use treatment programs should incorporate some form of behavioural therapy to teach patients to perceive the substance as unavailable, even when it may be accessible (Field and Cox, 2008b). The clinical relevance of attentional bias also extends to returning to smoking, as it appears to be a predictor of treatment outcomes among alcohol, heroin, cocaine, and tobacco users (Carpenter et al., 2006, Cox et al., 2007, Marissen et al., 2006, Waters et al., 2003a). In each of these cases, individuals showing greater attentional bias for a given drug-related cue had poorer treatment outcomes (Carpenter et al., 2006, Cox et al., 2007, Marissen et al., 2006, Waters et al., 2003a).

Reccurence of use is a major issue in the treatment of tobacco dependence, with most quit attempts ending in a return to smoking after only a few days (Clinical Practice Guideline Treating Tobacco Use and Dependence, 2008). Given this, there is growing interest in the role of attentional bias in the maintenance of smoking behaviour and vulnerability to recurrence of use (Christiansen et al., 2015, Drobes et al., 2019, Field et al., 2009). Multiple studies have reported relationships between attentional bias to smoking-related cues and increased probability of future substance use and returning to smoking (Bradley et al., 2003b, Field and Cox, 2008a, Hogarth et al., 2008, Robbins and Ehrman, 2004); however, findings are inconsistent (Christiansen et al., 2015). For example, greater bias in the Stroop Task has been found to be associated with both increased, and reduced likelihood of returning to smoking across studies (Spiegelhalder et al., 2011, Waters et al., 2003b). Further, these studies did not find any associations between attentional bias and returning to smoking when utilizing the Visual Probe Task (Spiegelhalder et al., 2011, Waters et al., 2003b). These discrepancies may stem from the predominant use of indirect methods to assess attentional bias to smoking-related cues, such as the aforementioned Stroop Task and Visual Probe Task (Drobes et al., 2006, Ehrman et al., 2002). The major limitation of both of these measures is that they infer attentional bias based on reaction times, making it difficult to ascertain whether the effects are due to attentional bias or impairments in motor response and cognitive ability (Eizenman et al., 2003). In recent years, eye-tracking technology has emerged as a more direct and reliable method of measuring attentional bias toward visual cues. The use of eye-tracking technology has shown efficacy in assessing visual orienting biases in various psychiatric disorders (e.g., depression, anxiety and eating disorders) and has also extended to substance use disorders (Armstrong and Olatunji, 2012, Bradley et al., 2016, Kerr-Gaffney, 2019, Maurage et al., 2020). Eye-tracking technology can produce several objective measures of attention that reflect early and late processes of attention, such as total fixation time (i.e., time spent looking at a stimulus), latency to fixation (i.e., time elapsed before gaze was directed to stimulus) and area of initial fixation (i.e., where gaze was first directed) (Drobes et al., 2019). Distinctions have been made between initial shifts in attention, which typically occur within 50–200 ms of stimulus onset, and the maintenance of attention (Bradley et al., 2003a, Ehrman et al., 2002). If attention is shifted to a stimulus within the first 200 ms of stimulus onset, this can reflect automatic, early orienting biases. When picture pairs are presented for longer durations (i.e., 2000 ms), this bias is more likely to reflect maintained attention (Bradley et al., 2003a, Ehrman et al., 2002).

Previous systematic reviews have investigated the use of eye-tracking indices for attentional bias in other substance use disorders, such as alcohol use disorder and cannabis use disorder (Bollen et al., 2021, Maurage et al., 2020, O'Neill et al., 2020); however, there has yet to be any comprehensive review investigating this topic in tobacco dependence. Other reviews have also explored attentional biases in people who smoke cigarettes, but primarily synthesized results using indirect measures of attentional bias (Field and Cox, 2008a, Robbins and Ehrman, 2004).

We conducted a comprehensive systematic review of existing literature examining attentional bias in people who smoke combustible tobacco cigarettes, measured using eye-tracking technology, to investigate: 1) differences in attentional bias to smoking-related cues among people who smoke cigarettes in comparison to people who don’t smoke and 2) differences in attentional bias to smoking-related cues in comparison to neutral cues among people who smoke cigarettes. By examining the contributions of attentional bias to smoking-related stimuli, we hope to provide meaningful insights into the theoretical understanding of tobacco dependence and help inform and enhance existing clinical interventions.

## 2 Methods

### 2.1 Article identification and selection procedure

The PICOS procedure (Population, Intervention, Comparator, Outcome, Setting) was used to determine the inclusion criteria (Liberati et al., 2009), as follows: 1) Population: only human studies were considered, and they had to include participants who were nicotine-dependent, and regularly smoked combustible tobacco cigarette as determined through a) number of cigarettes smoked per day or b) through standardized diagnostic tools (i.e. FTND) (Heatherton et al., 1991). Animal studies or studies that were not written in English were excluded. No exclusion was done based on participant demographics (i.e., age or sex) or previous/current psychiatric diagnosis. 2) Intervention: studies that used an eye-tracking device to assess attentional bias among people who smoke cigarettes were included. Studies that assessed attentional bias without an eye-tracking device were excluded from the review. 3) Comparator: studies were considered if they included a non-smoking group as a control group or if they compared smoking-related stimuli to neutral stimuli as a primary analysis. People who do not smoke were defined as those who have never smoked regularly (i.e., less than 100 cigarettes in a lifetime). 4) Outcome: studies were included if they proposed at least one eye-tracking index as a dependent variable (i.e., gaze preference, dwell time, fixation time). 5) Setting: studies were considered if they made comparisons between groups or experimental conditions (i.e., cross-sectional, interventional). Case studies or studies without experimental data were excluded.

### 2.2 Search strategy

**Fig. 1:**
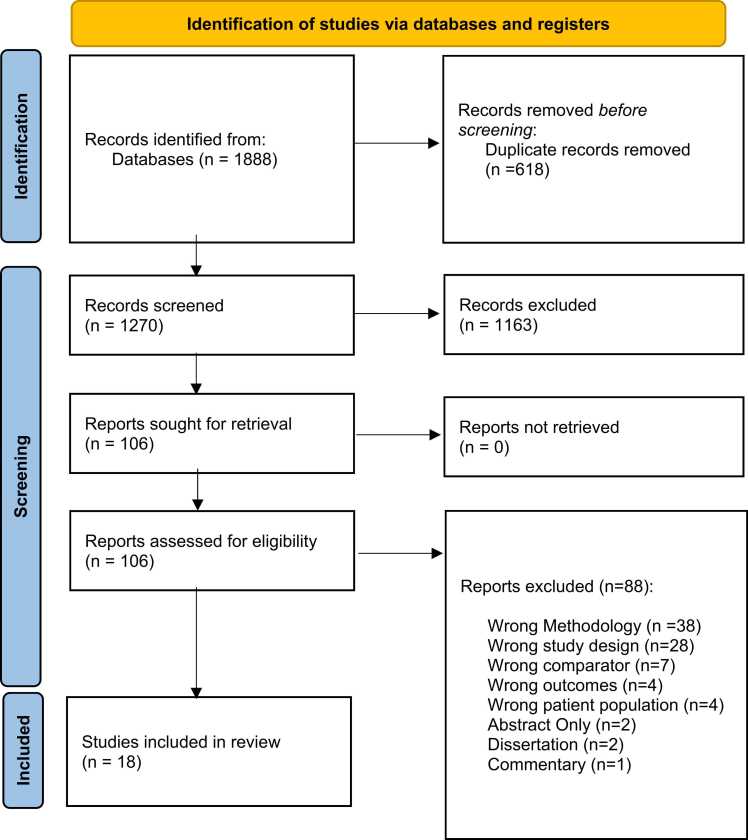
PRISMA flow diagram presenting the selection of the papers reviewed.

The Preferred Reporting Items for Systematic Reviews and Meta-Analyses guideline and related 27-item checklist were followed (Moher et al., 2009). A review of the literature on eye-tracking and attentional bias in people who smoke cigarettes was conducted using EMBASE, PsychInfo, and MEDLINE databases. Since eye-tracking is a newer technological measure, no restrictions were placed on publication dates. The final search of all databases occurred in October 2021. Every study that used eye-tracking indexes in people who regularly smoked cigarettes, without any limitations to participant demographics, medical history, sample size or outcome measures explored, was included. The following study designs were excluded from the review: case reports, reviews and meta-analyses, meeting abstracts/reports, editorials, comments, letters, guidelines, unpublished dissertations, and non-peer-reviewed literature. Search terms were in English only and combined eye-tracking (i.e. ((eye* or gaze or gazing or ocular or vision or visual) adj3 (display* or fix* or move or moving or movement* or orient* or search* or scan* or system* or stimul* or technology or technologies or test* or time* or timing or reflex* or track*)) and attentional bias (i.e. ((attention or cognit* or ocular or response or reaction or visual or vision) adj3 (bias* or cognit* or latency or latencies or preference* or process* or select* or time*)) and smoking (i.e. ((e-cig or e-cigarette or nicotine or tobacco or vape or vaping) adj3 (abuse* or addict* or consume* or consumption or dependen* or disorder* or use*)). For a more comprehensive list of terms, see **Appendix A**. The initial search identified 1888 papers. Search results were imported into a reference management software (The EndNote Team, 2013). The selection of the papers to be included followed a multi-step process **(see** Fig. 1**).** First, duplicates were identified and removed, leaving 1270 unique papers to be imported into a systematic review software (2022) and screened against title and abstract. Of the 1270 papers, 1163 studies were excluded due to lack of relevance to the study aims (i.e., no eye-tracking measure, no substance-related measure, no experimental data presented, and no non-human sample). This left 106 papers to be screened through full-text reading. Eighty-eight papers were excluded during this phase: 38 for wrong methodology (e.g., focus on health warnings), 28 for wrong study design (e.g., no eye-tracking), 7 for wrong comparator (e.g., use of emotional stimuli), 4 for wrong outcomes (e.g., cue reactivity outcomes), 4 for wrong patient population (e.g., patients with schizophrenia), 2 for abstract only, 2 were dissertations, and 1 was a commentary. This left 18 studies to be included in the review: 8 papers include a non-smoking control group as a comparator group, and 10 papers assess attentional bias differences within people who smoke cigarettes using an experimental control (neutral stimuli). The identification and selection procedures were carried out by the first and second authors (NR and AR, respectively). Regular discussions between the first and second authors took place to address any discrepancies in the selection of the articles. Any remaining disagreements were discussed with the senior author (LZ).

### 2.3 Methodological quality assessment

The methodological quality of each study was assessed using the "Quality Assessment Tool for Observational Cohort and Cross-Sectional Studies" developed by the National Heart, Lung, and Blood Institute (National Institutes of Health, 2014). This 14-item scale is widely used in various research fields, predominately to assess cross-sectional studies (Carbia et al., 2018, Koutsimani et al., 2019), and has been previously applied to systematic reviews with similar research questions (Maurage et al., 2021, Maurage et al., 2020).

This assessment scale had three answer choices: yes, no, or other (cannot determine, not applicable, or not reported). For each study, a score (i.e., percentage of items responded with a "yes") was calculated, leading to an overall quality rating (i.e., rated poor for scores of 0–4, fair for scores 5–10 and good for scores 11–14). Quality ratings were done by the first and second authors (NR and AR). Discussions between both authors resolved discrepancies in ratings. The methodological quality assessment was based on information provided in each paper; however, it should be noted that some criteria may have been considered in the study but not reported, which could affect the global rating. Therefore, these ratings should be considered as partially evaluating methodological quality rather than a conclusive rating on the quality of the paper.

### 2.4 Data extraction

A systematic data extraction was done for each paper for the following information: 1) general information (study ID, title, lead author contact details, country in which the study was conducted), 2) methods (aim of study, study design, conflicts of interest, type of instruments used to assess attentional bias, type of device used to track eye-movements), 3) participants (inclusion criteria, exclusion criteria, total number of participants, criteria used to define people who smoke, criteria used to define people who do not smoke), 4) results (attentional bias outcome used, eye-tracking outcome used), and 5) discussion (summary data for each group, key conclusions of the authors, study limitations). All eligible studies were separated into two sections based on the two research aims: studies that made comparisons between people who smoke and people who do not smoke (seen in Table 1) and studies that made comparisons between smoking and neutral stimuli among people who smoke (see Table 2).

**Table 1:** Description and main results of eye-tracking studies investigating attentional bias in people who smoke vs. people who do not smoke.

**Authors**	**Population**					
	**Sample Size (N)**	**Age (years)**	**Sex Ratio (%Male)**	**Inclusion/Exclusion Criteria**	**Criteria Used to Define People Who Smoke**	**Criteria Used to Define People Who Do Not Smoke**
Bonitz and Gordon (2008)	18 People who smoke 19 People who do not smoke	Not specified	People who smoke: 33 % People who do not smoke: 21 %	Corrected-to-normal vision	Not specified	Not specified
Bradley et al. (2007)	12 People who smoke 12 People who do not smoke	People who smoke: 25 years old People who do not smoke: 24 years old	People who smoke: 25 % People who do not smoke: 17 %	Corrected-to-normal vision English speaking Score of 10 or less on the Beck Depression Inventory-II	Smoked daily	Never having smoked regularly
Baschnagel (2013)	12 People who smoke 8 People who do not smoke	People who smoke: 21.6 (2.5) years old People who do not smoke: 20.6 (1.9) years old	People who smoke: 100 % People who do not smoke: 37.5 %	For satiated people who smoke: >6 ppm For people who do not smoke and abstinent people who smoke: ≥6 ppm	Smoked at least 10 CPD Not actively trying to quit smoking	Not smoked within past year Smoked less than 100 cigs in lifetime
Gamito et al. (2014)	21 People who smoke 34 People who do not smoke	21.65 (4.2) years old	Not specified	No brain injuries, cognitive impairments, or other psychiatric disorders	Moderate-to-highly dependent	Not specified
Kwak et al. (2007)	14 People who smoke 16 People who do not smoke	People who smoke: 22.5 (1.7) years old People who do not smoke: 20.9 (2.4) years old	People who smoke: 71 % People who do not smoke: 44 %	Corrected-to-normal vision	Not specified	Not specified
Lochbuehler et al. (2011)	16 People who smoke 17 People who do not smoke	People who smoke: 23.81 (10.14) years old People who do not smoke: 22.24 (8.87) years old	People who smoke: 56 % People who do not smoke: 18 %	Corrected-to-normal vision	Not specified	Never having smoked regularly
Mogg et al. (2003)	20 People who smoke 23 People who do not smoke	People who smoke: 23.1 (4.0) years old People who do not smoke: 23.6 (4.6) years old	People who smoke: 55 % People who do not smoke: 52 %	Corrected-to-normal vision English speaking For people who do not smoke: never smoked regularly	Not specified	Never having smoked regularly
Wilcockson et al. (2021)	34 Non-Dependent people who smoke 26 Dependent people who smoke 32 People who do not smoke	Non-Dependent people who smoke: 22.9 (7.4) years old Dependent people who smoke: 23.0 (7.6) years old People who do not smoke: 20.19 (4.0) years old	Non-Dependent people who smoke: 35 % Dependent people who smoke: 42 % People who do not smoke: 19 %	Not specified	Not specified	N/A

	**Design**			Outcomes			
**Authors**	**Type of Instrument Used to Track Eye-Tracking/ Sampling Rate**	**Stimuli**	**Eye-Tracking Indices**	**Main Results**	**Limitations**	**Key Conclusions**	**Methodological Quality**
Bonitz and Gordon (2008)	Eyelink Data Viewer 500 Hz	Consistent/neutral Inconsistent/neutral Consistent/smoking-related Inconsistent/smoking-related	Fixation Count Dwell Time Initial Fixation Duration	N.S. differences in fixation count, dwell time and initial fixations to smoking-related cues in people who smoke vs. people who do not smoke. However, people who smoke preferentially attend to smoking-related cues in comparison to people who do not smoke.	Completion of smoking questionnaire prior to eye-tracking task	Findings confirm the existence of attentional bias to smoking cues in people who smoke during viewing real-world scenes.	Poor
Bradley et al. (2007)	Pan/Tilt Optics System, Model 504	16 smoking-related cues 16 matched neutral cues	Direction of initial fixation Duration of Initial Fixation	People who smoke showed greater tendency to shift gaze toward smoking-related cues and greater urge to smoke when in a negative mood. People who smoke viewed smoking cues significantly longer than neutral cues regardless of mood states, whereas people who do not smoke viewed smoking cues longer in a negative mood only.	Small sample size	Findings offer partial support on the theory that negative mood increases attentional bias to drug cues and urge to smoke in people who smoke.	Fair
Baschnagel (2013)	Positive Science	2 smoking objects placed throughout room	Fixation Count Dwell Time	People who smoke made significantly more fixations to smoking cues than people who do not smoke. N.S. differences in dwell time	Sample was made of people who smoke lightly (low FTND score and # of cigs per day) People who smoke all male	Successful use of mobile eye-tracking for assessing attentional bias in people who smoke Smoking cues in the environment are more salient to people who smoke than people who do not smoke	Fair
Gamito et al. (2014)	Tobii-T60 60 Hz	Two virtual reality environments (high arousal cues and low arousal cues). Smoking cues present in high arousal condition	Fixation Count	Significantly greater fixations to smoking-related cues in people who smoke than in people who do not smoke.	Small sample size	Data supports the use of virtual environments for eliciting craving in people who smoke.	Fair
Kwak et al. (2007)	iView X Hi-Speed	4 smoking-related cues 2 aversive cues 2 neutral cues	Dwell time Direction of initial fixation	N.S. differences in direction of initial fixation between group. People who smoke showed longer gaze durations toward smoking-related cues than people who do not smoke. However, these findings were N.S.	Small sample size State-Trait Anxiety Inventory (STAI) administered after eye-tracking	Dwell time shown to be a sensitive measurement tool for identifying attentional bias.	Fair
Lochbuehler et al. (2011)	Tobii T120 60 Hz	43 minutes of the movie *Bridget Jones Diary* 58 smoking incidents in clip used	Fixation count Dwell time Latency of initial fixation	Significant differences in fixation counts, dwell time and latency of initial fixations to smoking-related cues in people who smoke compared to people who do not smoke.	No use of matched-control cues for comparison Sample not sex-matched Did not collect information on craving pre- or post-movie presentation	These findings indicate the effect of dynamic smoking cues (i.e., in movies), in addition to other environmental cues, and need to be taken into consideration in smoking cessation therapies.	Fair
Mogg et al. (2003)	Pan/Tilt Optics System, Model 504	20 smoking-related cues 20 matched neutral cues	Direction of initial fixation Duration of initial fixation Latency of initial fixation	N.S. differences in direction of initial fixation between group. People who smoke, but not people who do not smoke, looked longer and had shorter latencies to smoking-related pictures than people who do not smoke.	People who smoke were young, not heavily dependent, and more than half the sample smoked less than 20 cigs per day	People who smoke show biased attentional orientating to smoking cues, which is related to craving and the affective and motivational valence of the stimuli.	Fair
Wilcockson et al. (2021)	EyeLink 1000 1000 Hz	52 smoking-related cues 52 neutral cues	Fixation Count	Both dependent and non-dependent people who smoke had greater fixations toward smoking cues relative to neutral cues, while people who do not smoke had greater attentional bias to neutral cues than smoking cues.	Time since last cigarette not controlled for Participants did not abstain for a fixed amount of time prior to study Imbalanced group sizes	Increased attentional bias to substance-related cues may indicate continued substance usage.	Fair

**Table 2:** Description and main results of eye-tracking studies investigating attentional bias in people who smoke only.

**Authors**	**Population**				
	**Sample Size (N)**	**Age (years)**	**Sex Ratio (%Male)**	**Inclusion/Exclusion Criteria**	**Criteria Used to Define People who smoke**
Field et al., 2004b, Field et al., 2004a	23 People who smoke	21.96 (3.16) years old	57 %	Smoked at least 10 CPD Normally have the first cigarette before 11:00 AM English speaking Corrected-to-normal vision	Smoked at least 10 CPD
Field et al. (2005)	19 People who smoke	21.63 (2.93) years old	34 %	Daily cigarette smoking Consumption of at least 10 units of alcohol in the past week Consumption of at least four units of alcohol in a single session within past month Never received medical advice to quit/reduce drinking Body weight of at least 50 kg (females) or 60 kg (males) Corrected-to-normal vision	Smoked daily
Correa and Brandon (2016)	35 People who smoke	27.35 (4.72) years old	0 %	Ages 18–35 years old Smoked more than 100 cigarettes lifetime Smoked at least 5 cigarettes per day for the past 30 days Not currently attempting to quit smoking with counseling/pharmacotherapy No lifetime history of eating disorders Corrected-to-normal vision CO >6 ppm	CO>6 ppm Smoked more than 100 cigs in lifetime Smoked at least 5 CPD for past 30 days
Creswell and Skrzynski (2021)	90 People who smoke	38.4 (9.9) years old	52.2 %	Ages 18–50 years old Smoked ≥10 cigarettes/day for ≥12 months No interest in quitting English speaking No medical conditions that ethically contraindicated smoking Corrected-to-normal vision	Smoked at least 10 CPD for 12 months CO≥8 ppm
Haass-Koffler et al. (2021)	32 People who smoke	45.4 (11.8) years old	40.6 %	Ages 18–65 years old Enrolled to the Centre for Disease Control and Prevention (CDCFoundation) for heavy drinking (≥15 drinks/week for men; ≥8 drinks/week for women) Smoked at least 5 cigs/day No alcohol consumption the day of appointment No self-reported diagnosis of a severe SUD (except alcohol, caffeine, marijuana, or nicotine) No history of seizures Normal-to-corrected vision	Smoked at least 5 CPD
Kang et al. (2012)	25 People who smoke	25.0 (1.2) years old	100 %	Right-handed Free of any medication Smoked at least 10 CPD for over 3 years Corrected-to-normal vision No current diagnosis of psychiatric or neurological disorders	Smoked at least 10 CPD for over 3 years
Meng et al. (2014)	30 People who smoke	23.7 (7.2) years old	100 %	Ages 18–55 years old Smoked at least 8 CPD for at least 2 years Free of medication with active central nervous system properties No smoking cessation therapy history No history of major neurological disorders No brain injury or stroke history No vision diseases No chronic pain or hyperalgesia	Smoked at least 8 CPD for over 2 years
Mogg et al. (2005)	41 People who smoke	22.6 (4.9) years old	49 %	Smoke least 1 CPD English Speaking Corrected-to-normal vision	Smoked at least 1 CPD
Mondino et al. (2020)	22 People who smoke	41.8 (12.6) years old	64 %	Ages 20–60 years old Corrected-to-normal vision Not using any psychotropic medications No current or past diagnosis of a psychiatric disorder (excluding TUD) No contraindications to tACS (i.e., metal in the head, serious brain injury, etc.)	Smoke at least 15 CPD for at least 1 year Meet DSM−5 criteria for TUD Have a score of at least 4 on the FTND Desire to quit smoking in next 6 months Have made at least 1 quit attempt
Van Rensburg et al. (2009)	20 People who smoke	29.05 (9.37) years old	75 %	Ages 18–50 years old Smoked at least 10 CPD Regularly smoked for 2 years or more Not currently making a quit attempt Free from illness or injury	Smoked at least 10 CPD Regularly smoked for 2 years or more Not currently making a quit attempt

	**Design**			**Outcomes**			
	**Type of Instrument Used to Track Eye-Tracking/ Sampling Rate**	**Stimuli**	**Eye-Tracking Indices**	**Main Results**	**Limitations**	**Key Conclusions**	**Methodological Quality**
Field et al., 2004b, Field et al., 2004a	Pan/Tilt Optics System, Model 504	20 smoking-related cues 20 matched neutral cues	Direction of initial fixation Dwell time	When nicotine deprived, people who smoke maintained their gaze longer on smoking cues than neutral cues. This bias was reduced when non-deprived. People who smoke preferentially shifted gaze towards smoking cues in comparison to neutral cues. N.S. difference in initial fixations between conditions.	Small sample size No control group	Findings indicate that nicotine deprivation affects late attentional processes more than early attentional processes to smoking-related cues	Fair
Field et al. (2005)	Pan/Tilt Optics System, Model 504	20 smoking-related cues 20 matched neutral cues	Direction of initial fixation Dwell time	People who smoke maintained their gaze on smoking pictures for longer than neutral pictures The magnitude of this effect was larger after consumption of an alcoholic compared to a non-alcoholic drink. Alcohol had N.S effect on the initial shifting of gaze to smoking cues.	Small sample size Majority of participants were people who smoke lightly No evaluation of the extent to which the observed effects were due to alcohol expectancy effects vs pharmacological effects No attempt to mask taste of alcohol	Findings support theory that alcohol increases the incentive salience of smoking-related cues.	Fair
Correa and Brandon (2016)	Smart Eye Pro 60 Hz	20 test images categorized into four groups: Smoking/food Smoking/jewelry Food/jewelry Jewelry/jewelry	Dwell Time Direction of Initial fixations	Initial and maintained attention to smoking images was greatest when participants were administered a nonappetitive in vivo stimulus (jewelry).	Only female sample No control condition No assessment of nicotine withdrawal or hunger state Attentional bias to in vivo stimuli was not collected Did not collect information on key confounding variables (i.e., weight status, menstrual cycle phase)	Females who smoke preferred attending to smoking-related cues, and this preference was influenced by the presence of real-world environmental cues. These findings provide a step towards bridging gap between laboratory research and real-world experiences of females who smoke.	Fair
Creswell and Skrzynski (2021)	Pan/Tilt Optics System, Model ETPC-D6	20 colour photographs of smoking-related sciences 20 matched control pictures	Direction of Initial fixation Dwell time	N.S differences in dwell time between smoking-related cues and neutral cues, despite deprivation status. Significant differences were found in direction of initial fixations to smoking-related cues in whole group.	Was not powered to find significant associations when examining conditions separately or to detect significant differences across conditions	Maintenance of attention on drug-related cues may be a valid index of incentive motivation. However, dwell time may not be predictive of actual smoking behaviour.	Good
Haass-Koffler et al. (2021)	EyeLink 1000 1000 Hz	160 trials, with each trial showing pictures of water, alcohol and cigarette cues	Dwell Time	People who smoke spent a greater amount of time viewing alcohol and cigarette cues than water cues. N.S. differences in dwell time between alcohol and smoking cues.	Small sample size Not powered to separate into different smoking levels (light, moderate, and people who smoke heavily) Lack of administration of craving questionnaire in the eye-tracking procedure	Combining the presentation of cues in a virtual environment (eye-tracking) with a natural environment (cue-reactivity in bar laboratory) allows for a better understanding on the relationship between visual orienting and motivational valence of each cue presented simultaneously.	Fair
Kang et al. (2012)	iView XTMRED	15 smoking-related cues 15 matched neutral cues	Dwell time	Dwell time was longer in response to smoking-related cues than neutral cues Attentional bias to smoking cues measured in eye-tracking significantly correlated with brain activation in the dorsolateral prefrontal cortex (DLPFC), putamen, the posterior cingulate cortex, and the MI.	Only assessed one measure of attentional bias No control group Eye-tracking was done 30 min before fMRI, possible carry-over effects CO used to confirm 36-hour smoking abstinence	Attentional, motivational, and reward-related mechanisms play a role in smoking-related cue reactivity in people who smoke.	Fair
Meng et al. (2014)	Tobii Technology	Each picture consisted of 4 objects, one in each quadrant: 1 smoking-related cue 3 neutral cues	Fixation Count	Bilateral cathodal stimulation of the frontal-parietal-temporal association area reduced fixation to smoking-related cues; however, these effects were N.S.	Sample only male Number of cigarettes consumed was self-reported Only one session of transcranial direct current stimulation (tDCS)	Low current tDCS of the frontal-parietal-temporal association areas may have important clinical implications for smoking addiction treatment.	Good
Mogg et al. (2005)	Pan/Tilt Optics System, Model 504	20 smoking-related cues 20 matched neutral cues	Direction of initial fixation Dwell Time	People who smoke with lower levels of nicotine dependence showed longer dwell time to smoking-related cues. N.S differences in initial fixation time between smoking and neutral cues.	Main findings may be better explained by other variables, such as craving or time to last cigarette People who smoke were young Only small portion of people who smoke were heavily dependent Effect of craving and nicotine deprivation not examined independently	Severity of nicotine dependence was a negative predictor of attentional biases and craving was a positive predictor of attentional biases.	Fair
Mondino et al. (2020)	Viewpoint Eye-Tracker	16 trials that include 1 smoking-related image and 3 neutral images in one frame	Dwell Time	Active transcranial alternating current stimulation (tACS) combined with attentional bias modification (ABM) reduced the amount of time spent looking at smoking-related cues, prevented an increase of self-reported desire to smoke, and reduced proportion of impulsive choices compared to sham tACS + ABM.	Issues with successful blinding No condition investigating the effect of alpha-tACS without ABM One single tACS session	Combining tACS with ABM may help people who smoke who wish to quit by reducing the desire to smoke, reducing attention to smoking-cues and impulsive decision making.	Fair
Van Rensburg et al. (2009)	EyeLink II 500 Hz	20 smoking-related cues 20 matched neutral cues	Dwell Time Direction of Initial Fixation	Dwell time and direction of initial fixations towards smoking images were reduced significantly following the exercise condition compared with passive control condition.	Only used two measures of attentional bias Assessed only a single session of exercise at one level of intensity Study was done in a laboratory	Single session of exercise can reduce the desire to smoke and influence two measures of attentional bias toward smoking images.	Fair

## 3 Results

### 3.1 General study characteristics

A total of 18 published studies were included in this review. The general geographic distribution of the studies selected (affiliations of the first author) was 34 % in North America (5 in the United States and 1 in Canada), 50 % in Europe (6 in the United Kingdom, 1 in the Netherlands, 1 in Belgium and 1 in Portugal), and 17 % in Asia (2 in South Korea and 1 in China). Publication dates suggest an increase in publications using eye-tracking to assess attentional bias in people who smoke within the last decade, with 42 % of these studies published between 2000 and 2010 and 58 % published between 2011 and 2021. Most of the study participants were young adults, with 79 % of published studies having participants with a mean age of 20–30. Only 16 % of studies had participants with a mean age of 30–50. One study did not report on the mean age of their study participants. Many of the studies included were cross-sectional (78 %). Some studies provided an intervention such as alcohol, transcranial direct current stimulation (tDCS), transcranial alternating current stimulation (tACS) or acute exercise. Many studies (42 %) combined reaction-based behavioural tasks (i.e., Visual Probe Task) and eye-tracking measures to assess attentional bias. Lastly, eye-tracking indices most used were dwell time (72 %), direction of initial fixation (42 %), fixation count (37 %), duration of initial fixation (21 %), and latency of initial fixation (11 %).

### 3.2 Quality assessment

Of the 18 studies evaluated for methodological quality, 1 of the studies was considered "poor" quality, 15 were considered "fair" quality, and 2 were rated "good" quality (Table 1 and 2). The strengths of the studies assessed included clear statements of research objectives and the implementation of a well-designed methodology. The vast majority of studies selected made controlled comparisons between smoking group (people who smoke vs. people who do not smoke) or stimulus type (smoking vs. neutral stimuli); however, three studies used an intervention (i.e., tDCS, tACS, acute exercise) to mitigate attentional biases in people who smoke (Meng et al., 2014, Mondino et al., 2020, Van Rensburg et al., 2009). Moreover, most studies implemented reliable and valid eye-tracking indices to assess attentional bias, such as fixation count and dwell time. All studies reported the brand and type of the eye-tracking device used, and the models used are considered valid. However, many of the studies did not report the sample rate of the eye-tracking device, and of those that did, large variations were found (60 Hz to 1000 Hz). Such large variations limit inter-study comparability. Furthermore, the main limitations of these studies were the lack of justification for the sample size calculation and the absence of power and effect size calculations to estimate the strength of their findings. Additionally, many studies had small sample sizes, and did not control for key variables, such as time to last cigarette or number of cigarettes smoked per day (few of the studies acknowledged that their sample comprised mainly of people who smoke lightly). Lastly, almost all participant samples were recruited from the general population, with limited inclusion/exclusion criteria outlined. Therefore, there was low control of population characteristics, and a large portion of these studies did not account for variables that could have influenced eye-tracking and attentional bias (i.e., psychiatric comorbidities, mood, and polysubstance use/medications).

### 3.3 Main outcomes

Studies were grouped and analyzed as a function of attentional processes investigated (i.e., early orienting vs. attentional maintenance) and type of comparison (i.e., people who smoke vs. people who do not smoke, within people who smoke only). Some studies used multiple measures of attentional maintenance, early processes of attention, or both. Of the 18 studies selected for review, 8 studies used eye-tracking measures to examine differences in attentional bias between people who smoke (N=173) and people who do not smoke (N=161), see Table 1. Of these, six studies used attentional maintenance as an outcome measure of interest (fixation count was used in 5 studies (Baschnagel, 2013, Bonitz and Gordon, 2008, Gamito et al., 2014, Lochbuehler et al., 2011, Wilcockson et al., 2021), dwell time used in 4 studies (Baschnagel, 2013, Bonitz and Gordon, 2008, Kwak et al., 2007, Lochbuehler et al., 2011). Five studies used early processes of attention as an outcome measure of interest (direction of initial fixation was used in 4 studies (Bonitz and Gordon, 2008, Bradley et al., 2007, Kwak et al., 2007, Mogg et al., 2003), duration of initial fixation was used in 2 studies (Bradley et al., 2007, Mogg et al., 2003), and latency of initial fixation was used in 2 studies (Lochbuehler et al., 2011, Mogg et al., 2003).

Further, 10 studies examined differences in attentional bias to smoking cues and neutral cues among people who smoke (N=337); see Table 2. Of these, attentional maintenance to smoking-related cues was indexed by fixation count in 1 study (Meng et al., 2014), and dwell time in 9 studies (Correa and Brandon, 2016, Creswell and Skrzynski, 2021, Field et al., 2004a, Field et al., 2005, Haass-Koffler et al., 2021, Kang et al., 2012, Mogg et al., 2005, Mondino et al., 2020, Van Rensburg et al., 2009). Six studies examined early attentional allocation by measuring the direction of initial fixation (Correa and Brandon, 2016, Creswell and Skrzynski, 2021, Field et al., 2004a, Field et al., 2005, Mogg et al., 2005, Van Rensburg et al., 2009). Due to the different attentional processes and research questions being studied, results from these studies are presented separately.

### 3.4 Attention maintenance in people who smoke vs. people who do not smoke: fixation count

Five studies used fixation count as one of the primary outcome variables of interest (Baschnagel, 2013, Bonitz and Gordon, 2008, Gamito et al., 2014, Lochbuehler et al., 2011, Wilcockson et al., 2021). Fixation counts are a measure of the number of fixations within an area of interest. In all five of the studies, people who smoke showed greater bias towards smoking-related cues than people who do not smoke. However, only four of the five studies found this difference between groups to be statistically significant. One of these studies, Baschnagel (2013), asked people who smoke to complete two sessions of eye-tracking: one satiated and one after 12-hour smoking abstinence. No significant differences in fixation counts were observed between people who smoke that were abstinent and satiated.

Gamito et al. (2014) investigated differences in number of fixations to smoking-related cues in a virtual reality environment in 72 undergraduate students (people who smoke: n=21, people who do not smoke: n=34). They found that the number of eye fixations to smoking-related cues was significantly higher in people who smoke than in people who do not smoke. Similarly, Lochbuehler et al. (2011) examined differences in attentional bias in people who smoke (n=16) and people who do not smoke (n=17) for dynamic cues during a 43-minute clip of a contemporary movie ("*Bridget Jones Diary"*). Researchers found that people who smoke had significantly greater fixations to smoking-related cues in the movie clip than people who do not smoke. Moreover, in another study, Baschnagel (2013) tested the use of a mobile eye-tracking procedure to assess attentional bias to smoking-related cues in a more naturalized environment (i.e., a research assistant’s office space) in people who smoke (n=12) and people who do not smoke (n=8). People who smoke were asked to complete two sessions: one satiated and one after 12-hour smoking abstinence. Findings revealed significantly more fixations to smoking-related cues in people who smoke (both when abstinent and non-abstinent) than in people who do not smoke. No significant differences in fixation counts were observed between people who smoke that were abstinent and satiated. Furthermore, Bonitz and Gordon (2008) examined attentional bias to smoking-related stimuli during the presentation of real-world photographic scenes, such as an office desk with either chewing gum (neutral scene) or a cigarette pack (smoking-related scene) placed on top. People who smoke (n=18), but not people who do not smoke (n=19), had significantly greater fixations to smoking-related objects than neutral objects. However, differences in attentional bias between groups did not reach statistical significance. Lastly, Wilcockson et al. (2021) used the eye-tracking version of the standard dot-probe task (Field et al., 2016) to assess differences in attentional bias to smoking-related cues in people who smoke that were dependent (n=26), people who smoke that were non-dependent (n=34) and people who do not smoke (n=32). Researchers found that fixation counts to neutral stimuli were significantly greater than fixation counts to smoking-related stimuli for people who do not smoke. Contrarily, for both dependent and non-dependent people who smoke, there were significantly greater fixation counts to smoking-related stimuli in comparison to neutral stimuli. Significant group differences were also observed, whereby people who do not smoke and people who smoke that were non-dependent significantly differed in fixations counts to smoking-related stimuli. No differences were found between non-dependent and dependent people who smoke. In summary, while only one study did not find statistically significant differences between groups on fixation counts to smoking-related cues, all studies showed greater bias towards smoking-related cues in people who smoke than in people who do not smoke.

### 3.5 Attention maintenance in people who smoke vs. people who do not smoke: dwell time

Dwell time is defined as the mean length of time (in ms) that eye fixations are confined to a specific visual stimulus (Vansteenkiste et al., 2015). Longer durations of looking at a certain area of interest indicate high level of interest, while shorter durations indicate lower levels of interest. Four studies examined differences in dwell time to smoking-related cues between people who smoke and people who do not smoke (Baschnagel, 2013, Bonitz and Gordon, 2008, Kwak et al., 2007, Lochbuehler et al., 2011). Only one study found significant differences in dwell time between people who smoke and people who do not smoke (Lochbuehler et al., 2011). In this study, people who smoke directed their gaze significantly longer to smoking-related cues than people who do not smoke (Lochbuehler et al., 2011). In all other studies, no statistically significant differences were found between groups (Baschnagel, 2013, Bonitz and Gordon, 2008, Kwak et al., 2007). However, while not statistically significant, Kwak et al. (2007) found that people who smoke (n=14) spent a greater amount of time viewing smoking-related cues than people who do not smoke (n=16). Similarly, while no between-group differences were found, Bonitz and Gordon (2008) found that people who smoke spent significantly longer looking at smoking-related cues relative to neutral cues. For people who do not smoke, dwell time between stimuli types did not statistically differ.

### 3.6 Early processes of attention in people who smoke vs. people who do not smoke: direction, duration and latency of initial fixations

Direction, duration, and latency of initial fixations are indices of early attentional allocation (Colombo and Mitchell, 1990). Direction, duration and/or latency of initial fixations were reported in five studies in total: four examined the direction of initial fixation (Bonitz and Gordon, 2008, Bradley et al., 2007, Kwak et al., 2007, Mogg et al., 2003), two examined the duration of initial fixation (Bradley et al., 2007, Mogg et al., 2003) and two examined the latency of initial fixation (Lochbuehler et al., 2011, Mogg et al., 2003).

For direction of initial fixation, most findings were not statistically significant. Specifically, Kwak et al. (2007), Bonitz and Gordon (2008), and Mogg et al. (2003) found no significant differences between people who smoke and people who do not smoke on the direction of initial fixation toward visual cues. However, both Kwak et al. (2007) and Mogg et al. (2003) found that in comparison to the 50 % threshold, which indicates no bias toward smoking-related cues relative to neutral cues, people who smoke directed their gaze to smoking-related visual cues on more trials than people who do not smoke (51.1 % vs 48.8 % of trials, respectively in Kwak et al., 2007; 54 % vs. 50.4 % of trials, respectively in Mogg et al., 2003).

Bradley et al. (2007) investigated the effect of negative mood on attentional bias to smoking-related cues by experimentally inducing a negative or neutral mood in counterbalanced order on separate sessions before the eye-tracking task. Interestingly, researchers found that people who smoke were significantly more likely to direct their gaze initially toward smoking-related cues when in a negative mood in comparison to when in a neutral mood. In contrast, no differences were found in people who do not smoke on initial orienting bias, irrespective of mood. Additionally, they found that increased negative mood was associated with greater initial orienting bias in people who smoke, but not in people who do not smoke (Bradley et al., 2007).

Duration of initial fixation can be interpreted as the extent of initial attentional allocation (Colombo and Mitchell, 1990). Mogg et al. (2003) found that people who smoke (n=20) had longer fixation durations toward smoking-related cues than neutral cues, whereas people who do not smoke (n=23) showed no significant differences in initial fixation durations for smoking-related cues and neutral cues. Similarly, Bradley et al. (2007) found that people who smoke (n=12) had significantly longer initial fixations to smoking-related cues than neutral cues, in both negative and neutral mood-induced conditions. On the other hand, people who do not smoke (n=12) had significantly longer initial fixations to smoking-related cues only when in a negative mood.

The latency of initial fixations is defined as the time interval (in ms) between cue onset and the fixation toward the cue (Colombo and Mitchell, 1990, Mogg et al., 2003). Two studies examined differences in latency of initial fixations between people who smoke and people who do not smoke to smoking-related cues. Lochbuehler et al. (2011) found that during a 43-minute segment of a contemporary movie, people who smoke directed their gaze more quickly toward smoking-related dynamic cues than people who do not smoke. Similarly, Mogg et al. (2003) found that people who smoke had shorter latencies for each picture type (smoking-related cues and neutral cues) than people who do not smoke.

### 3.7 Attention maintenance in people who smoke: fixation count

Meng et al. (2014) used fixation count as the primary outcome measure of interest to assess attentional bias to smoking-related cues. In this study, participants (n=30) were randomly assigned to receive either 1) a single tDCS stimulation with the cathode electrode on the right frontal-parietal-temporal association (FTP) area, 2) bilateral tDCS stimulation with the cathode on both sides of the FTP or 3) sham-tDCS. In the whole group, significantly greater fixations towards smoking-related cues were found in comparison to neutral cues prior to the start of treatment. While they found no statistically significant differences between stimulation groups after treatment, post-hoc tests showed that bilateral stimulation reduced smoking-related attentional bias, as indicated by fewer fixation counts toward smoking-related cues. No other significant main effects or interactions were found (Meng et al., 2014).

### 3.8 Attention maintenance in people who smoke: dwell time

A total of 9 studies used dwell time as an index of attentional bias. Only one study (Creswell and Skrzynski, 2021) failed to find a significant difference in dwell time to smoking-related pictures in comparison to neutral pictures, irrespective of whether they were nicotine-deprived (12-hour smoking abstinence) or non-deprived (n=90). Field et al. (2005) found that participants (n=19) viewed smoking-related cues significantly more than neutral cues, regardless of whether participants consumed an alcoholic beverage prior to the eye-tracking task or not. However, they found that the difference in dwell time to smoking-related cues relative to neutral cues was far greater after the consumption of an alcoholic beverage in comparison to a non-alcoholic beverage. Haass-Koffler et al. (2021) simultaneously presented an alcohol-related cue, a smoking-related cue and a neutral cue and found that people who smoke (n=32) spent a significantly greater time looking at alcohol-related and smoking-related cues than neutral cues. No differences were found in dwell time between smoking-related cues and alcohol-related cues among people who smoke. Furthermore, Mogg et al. (2005) investigated attentional bias to smoking-related cues as a function of varying levels of nicotine dependence. Participants (n=41) were assigned to either low or moderate dependence groups based on whether their FTND scores fell below the median of 3. Findings revealed that in the whole group, people who smoke looked significantly longer at smoking-related cues in comparison to neutral cues. However, when comparing groups, the low-dependence group exhibited significantly longer dwell times toward smoking-related images compared to the moderate-dependence group. The two groups did not significantly differ in dwell time toward neutral cues.

Similarly, Kang et al. (2012) found significant differences in dwell time to visual stimuli, whereby people who smoke (n=25) spent significantly longer looking at smoking-related cues in comparison to neutral cues. Interestingly, they found that increased attentional bias to smoking-related cues correlated with subjective craving, as well as brain reactivity in areas involved in executive functioning and attentional motivation. Field et al. (2004a) examined the effect of nicotine deprivation on attentional biases for smoking-related cues. Participants (n=23) were instructed to attend two study sessions, a nicotine-deprived session and a non-deprived session, at least one week apart. Before attending the non-deprived session, participants were instructed to maintain their normal smoking behaviour and to have a cigarette immediately before arriving at the laboratory. Before attending the deprived session, participants were directed to refrain from smoking for at least 10 hours. In the whole group, participants viewed smoking-related cues significantly more than neutral cues. Researchers also found that the difference in dwell time between smoking-related cues and neutral cues was significantly greater in the deprived condition than the non-deprived condition.

Correa and Brandon (2016) investigated differences in attentional bias to smoking-related cues against a nonappetitive neutral cue (jewellery) and an appetitive neutral cue (food) in 35 females who smoke cigarettes. They also compared how the presence of an in-vivo stimuli (cigarettes, food, and jewellery) during the eye-tracking task affected the attentional bias to these cues. Regardless of the in-vivo condition, participants displayed greater attentional bias, as indexed by greater dwell times, to smoking-related cues than both neutral cues. However, when examining the effect of the in-vivo stimulus condition on attentional bias, participants demonstrated significantly longer gaze durations toward smoking-related cues than jewellery- and food-related cues in the jewellery in-vivo condition. Additionally, when smoking and food cues were presented simultaneously, participants viewed smoking-related cues longer than food cues in the in-vivo jewellery condition.

Van Rensburg et al. (2009) examined the impact of acute exercise on attentional bias to smoking-related cues following a 15-hour period of nicotine abstinence. In a randomized cross-over design, people who smoke regularly (n=20) engaged in 15 minutes of moderate-intensity stationary cycling or passive seating on separate days. Researchers found a significant increase in dwell time to smoking-related cues from baseline to post-treatment in the passive condition and a significant decrease in dwell time to smoking-related cues from baseline to post-treatment in the exercise condition. Additionally, in post-treatment, dwell time to smoking-related cues was significantly reduced in comparison to neutral cues following exercise relative to passive seating. Lastly, Mondino et al. (2020) investigated the effectiveness of attentional bias modification in combination with tACS applied over the dorsolateral prefrontal cortex (DLPFC) on attentional bias to smoking-related cues in people who smoke (n=19). They found that active tACS + attentional bias modification significantly reduced time on the smoking-related quadrant in comparison to those receiving sham tACS + attentional bias modification.

### 3.9 Early attentional processes in people who smoke: direction of initial fixation

Six studies examined early visual orienting bias to smoking-related cues in people who smoke. Creswell and Skrzynski (2021) examined differences in attentional bias to smoking-related cues in people who smoke who were either nicotine-deprived or non-nicotine-deprived. They found that despite nicotine deprivation status, participants showed significant attentional bias for smoking-related cues, as indexed by initial fixation bias scores. No significant differences were observed between conditions. Four studies (Van Rensburg et al., 2009, Mogg et al., 2005; Field et al., 2004; Field et al., 2005) assessed initial gaze direction bias to smoking-related cues by comparing bias scores to 50 %. Bias scores of less than 50 % indicate a bias towards neutral cues, whereas a bias of 50 % indicates no bias. First, Field et al. (2004a) observed that people who smoke directed their gaze toward smoking scenes in 55.3 % of trials in the deprived condition and 55.7 % of trials in the non-deprived condition, with no significant differences being found between conditions. When investigating whether people who smoke showed a preference for directing their gaze towards smoking-related images over control images, they found a significantly greater percentage of fixations on smoking-related cues in both the deprived and non-deprived conditions.

Second, Van Rensburg et al. (2009) found that after acute exercise, people who smoke directed their gaze toward smoking-related cues in 53.5 % and 43.0 % of trials at baseline and after 15 minutes of moderate-intensity cycling, respectively, while those in the passive seating condition directed their gaze towards smoking-related cues on 51.3 % and 54.8 % of trials at baseline and after 15 minutes of passive sitting. No differences were found between baseline and post-treatment bias scores in the passive seating condition. However, significant differences between baseline and post-treatment scores in the exercise condition were found, as well as significant differences between post-treatment scores in the control and exercise conditions. Third, Mogg et al. (2005) found that people who smoke that had low nicotine-dependence made their first fixation to smoking-related pictures on 53.5 % of the trials. In contrast, the moderate dependent group made their first fixation to the smoking pictures on 51.2 % of the trials. While no significant differences were found between the two conditions, the low dependence group, but not the moderate dependence group, had significantly greater fixation to smoking-related cues than neutral cues.

On the other hand, Field et al. (2005) investigated whether alcohol would increase attentional bias to smoking-related cues and found no significant differences in the direction of initial fixation between those who consumed an alcoholic beverage and those who did not prior to the eye-tracking task. However, they found that after the consumption of alcohol, people who smoke directed their gaze at smoking-related cues on 54.6 % of trials, while those who consumed a non-alcoholic beverage directed their gaze at smoking-related cues on 52.1 % of trials. Neither of these findings were significantly different from 50 %. Lastly, in Correa and Brandon's (2016) study, participants showed increased initial fixation towards smoking-related cues than appetitive neutral (food) and nonappetitive neutral (jewellery) cues across all conditions (smoking, food, and jewellery in-vivo). Additionally, when smoking-related cues and appetitive neutral cues were presented simultaneously, participants had an increased number of initial fixations toward smoking-related cues than neutral cues.

## 4 Discussion

This systematic review provides a qualitative summary of the existing literature examining attentional bias in people who smoke using eye-tracking indices. We identified 8 studies that compared attentional bias between people who smoke and people who do not smoke using eye-tracking indices **(**Table 1**)**, and 10 studies that investigated attentional bias to smoking-related cues among people who smoke **(**Table 2**)**. Many of the studies reviewed included both early (i.e., duration of initial fixation, direction of initial fixation, etc.) and late (i.e., dwell time and fixation counts) indices of attentional processes as outcome measures of interest. Although evidence for attentional bias was consistent across both study designs, their methodological differences are important to consider. Studies that utilized group-based comparisons between people who smoke and those that do not smoke primarily aimed to establish the existence of an enhanced attentional bias to smoking cues among people who smoke, providing evidence for attentional bias as a potential cognitive marker of tobacco dependence. These studies are also used to establish the feasibility of specific eye-tracking paradigms and cue sets for future research by determining whether they reliably detect attentional bias in people who smoke compared to those who do not. In comparison, studies focused exclusively on people who smoke typically assessed how attentional bias was influenced by different conditions, such as nicotine deprivation or following an intervention. These studies offer insights into the dynamic nature of attentional bias under varying conditions, making them particularly useful for evaluating treatment effects and understanding state-dependent changes. Both methodologies contribute uniquely to understanding the role of attentional bias in tobacco use. Therefore, future eye-tracking studies should carefully align their methodologies with their specific research objectives.

We found that people who smoke had significantly greater attentional bias to smoking-related cues, as indexed by greater dwell times and fixation counts. However, findings using measures of early orienting biases were mixed but showed a tendency to initially shift attention to smoking-related cues more often than neutral cues. Findings from the summarized studies suggest that maintained attention may provide a more precise representation of the attentional mechanisms that are influenced by incentive salience, particularly, the bias to maintain attention towards motivationally salient visual cues (Field et al., 2004b). Other attentional processes, such as early attentional shifts, may be less sensitive to the type of attentional bias found in substance use disorders. Interestingly, biases in the maintenance of attention, but not initial shifts in attention, have been found to correlate with subjective craving (Field et al., 2004b). Measures of dwell time or fixation count may provide an extremely useful and objective index of the strength of incentive motivational processes involved, which are shown to play a predictive role in drug-taking behaviour and early recurrence of use (Bradley et al., 2003b, Hogarth et al., 2009, Hogarth et al., 2007). One of the major advantages of eye-tracking technology to assess attentional bias is that it does not rely on reaction times, self-reports or introspection; thus, it provides a more reliable measurement of sub-conscious incentive motivational processes (Marks et al., 2014, Skinner et al., 2018).

### 4.1 Attention maintenance: comparisons between people who smoke vs. people who do not smoke and among people who smoke

Fixation count and dwell times are highly correlated and directly observable metrics of attentional maintenance (Hogarth et al., 2009). Both dwell time and fixation counts measure the time at which an individual's attention is devoted to an area of interest. Longer dwell times or higher fixation counts suggest that participants require more cognitive capacity for processing information from a specific visual stimulus (Kelley et al., 2008). Many of the studies reviewed provided evidence for increased maintenance of attention to smoking-related cues in people who smoke in comparison to non-smoking controls. More specifically, most studies found higher fixation counts to smoking-related cues in people who smoke in comparison to people who do not smoke (Baschnagel, 2013, Gamito et al., 2014, Lochbuehler et al., 2011, Wilcockson et al., 2021). Only one study did not find statistically significant differences but found higher fixation counts to smoking-related objects in comparison to neutral objects in people who smoke but not in people who do not smoke (Bonitz and Gordon, 2008). One potential explanation for the absence of significant group differences in this study could be attributed to the participants’ completion of a questionnaire on smoking habits prior to the eye-tracking task, potentially priming both people who smoke and people who do not smoke to selectively attend to smoking-related objects (Bonitz and Gordon, 2008). However, despite the possibility of a priming effect, people who smoke (in contrast to people who do not smoke) displayed significant attentional bias to smoking-related objects compared to neutral objects, indicating the strength of the maintenance of attention. Interestingly, studies examining dwell time as a measure of maintenance of attention found mixed findings. Only one study found statistically significant differences in gaze duration to smoking-related cues between groups (Lochbuehler et al., 2011). Studies by Baschnagel (2013), Kwak et al. (2007) and Bonitz and Gordon (2008) found longer gaze duration toward smoking-related cues in people who smoke in comparison to people who do not smoke; however, results did not reach statistical significance. It is possible that the small sample size in all three studies (Baschnagel., 2013, N=20; Kwak et al., 2007, N=30; and Bonitz and Gordon, 2008, N=37) resulted in insufficient power to detect group differences. Despite this, findings still revealed a greater amount of time viewing smoking-related cues in people who smoke than in people who do not smoke. Larger studies are required to compare dwell time using eye-tracking methodology in people who smoke vs. people who do not smoke.

When exploring indices of attentional maintenance among people who smoke, one study used fixation count and eight studies used dwell time as the primary outcome measure of interest. Results were consistent for both fixation counts and dwell time: people who smoke fixated more and spent longer viewing smoking-related cues than neutral cues. Interestingly, many of these studies used various experimental conditions to explore factors that may contribute to the strength of the maintenance of attention in people who smoke. First, Field et al. (2005) found that dwell time toward smoking-related cues increased after the consumption of an alcoholic beverage in comparison to a non-alcoholic beverage, suggesting that alcohol may selectively increase the maintenance of attention, as well as increase the incentive salience of smoking-related cues. Further, attentional bias toward smoking-related cues was increased in people who smoke with lower dependence (Mogg et al., 2005) and those experiencing nicotine deprivation (Field et al., 2004a). Consistent with previous studies using indirect measures (i.e., reaction-based tasks), some studies indicate that attentional bias towards smoking-related cues is more pronounced in light people who smoke and in individuals experiencing heightened subjective nicotine craving (Bradley et al., 2003b, Field and Cox, 2008b, Waters et al., 2003a). It has been hypothesized that people who smoke with low levels of nicotine dependence show heightened attentional and approach biases for smoking-related cues, possibly due to a transition from "incentive responding" to "habit-based responding" (Patrono et al., 2017). However, this theory has been challenged by other studies showing greater attentional bias to smoking-related cues in more frequent, dependent people who smoke (Wilcockson et al., 2021). More research is needed to examine how addiction progression impacts attentional bias to smoking-related cues, particularly with the use of more direct methods, such as eye-movement data.

Further, a study by Correa and Brandon (2016) found that dwell time to smoking-related cues was significantly greater when in the presence of a nonappetitive in-vivo neutral stimulus in comparison to an appetitive in-vivo stimulus. Findings from this study suggest that attentional bias to smoking-related cues could be influenced by the presence of appetitive in-vivo stimuli. Such stimuli may decrease the motivation to focus attention on other environmental cues associated with smoking, particularly when there is anticipation of consuming the in-vivo stimulus (Correa and Brandon, 2016). Finally, three studies presented a treatment-based study design aimed to assess whether a specific intervention could reduce the maintenance of attention to smoking-related cues. Meng et al. (2014) found that compared to people who smoke who received sham-tDCS, bilateral tDCS stimulation on the frontal-parietal-temporal association area reduced smoking-related attentional bias and significantly reduced daily cigarette consumption. Similarly, Mondino et al. (2020) found that active tACS stimulation, combined with attentional bias modification, significantly reduced attentional time spent viewing smoking-related cues and prevented the increase of self-reported desire to smoke compared to those receiving sham tACS + attentional bias modification. Moreover, Van Rensburg et al. (2009) found that 15 minutes of moderate-intensity stationary cycling significantly decreased dwell time to smoking-related cues and self-reported desire to smoke compared with the passive seating condition. The results of these studies suggest that targeting aberrant attentional maintenance to smoking-related cues can alter smoking behaviour, as seen in reductions in cigarette consumption and self-reported desire to smoke. However, future studies are warranted to confirm the direction of findings. In conclusion, the findings from the summarized studies above suggest that attentional maintenance is a robust effect observed among people who smoke, irrespective of the experimental condition used. Consistent with Robinson and Berridge's (1993), (2003) incentive-sensitization theory, findings show that people who smoke significantly engage and sustain their attention to smoking-related cues compared to neutral cues.

### 4.2 Early processes of attention: comparisons between people who smoke vs. people who do not smoke and among people who smoke

Initial shifts in attention reflect fast, preconscious, and automatic shifts in attention and have shown to play an important role in mediating attentional biases (Mogg et al., 1997). One of the methods used in many of the studies assessing the direction of initial fixation was calculating the percentage of trials the initial fixation was on the smoking-related cue (Bradley et al., 2007, Mogg et al., 2003). This percentage was statistically compared to 50 %, which reflected equal bias between both cues. A percentage of less than 50 % indicated bias to the neutral cue. Studies comparing differences in people who smoke and people who do not smoke on the direction of initial fixations mainly had negative findings, with the majority of the studies finding no significant differences between groups (Bonitz and Gordon, 2008, Kwak et al., 2007, Mogg et al., 2003). However, while not statistically significant, all studies reviewed showed people who smoke preferentially directing their gaze toward smoking-related cues, while people who do not smoke showed no bias or a bias toward neutral cues. Only one study found significant differences between groups on the direction of initial fixation, where increased negative mood was associated with greater initial orienting bias in people who smoke, but not in people who do not smoke (Bradley et al., 2007). Additionally, they found that people who smoke were significantly more likely to direct their gaze initially toward smoking-related cues when in a negative mood in comparison to when in a neutral mood. Differential findings in the studies above support the affective processing model, by Baker et al. (2004), which suggests that negative mood influences early aspects of visual processing. Baker et al. (2004)'s model proposes that early processes of attention are sensitive to the effects of mood and that negative mood increases the incentive salience of drug cues. On the other hand, later aspects of attentional processing, such as the maintenance of attention, are thought to be less sensitive to mood effects, given that non-affective information is incorporated into the processing of visual cues at this stage, diluting the strength of the affective signal (Baker et al., 2004). Thus, the lack of controlling for important variables, such as mood, may have influenced the findings. Future studies should examine the moderating effects of negative mood on different aspects of attentional-processing biases for smoking-related cues.

Furthermore, the duration of initial fixation and latency of initial fixation can also be used as an index of early attentional processing (Colombo and Mitchell, 1990). Two of the reviewed studies used duration of initial fixation, both of which found that people who smoke had longer initial fixation durations to smoking-related cues than neutral cues (Bradley et al., 2007, Mogg et al., 2003). This difference was not observed in people who do not smoke. Similarly, latency of initial fixations was examined in two studies, and findings revealed that people who smoke had shorter latencies to smoking-related cues than people who do not smoke (Lochbuehler et al., 2011, Mogg et al., 2003). These additional measures lend some support for differences in early attentional processes between people who smoke and people who do not smoke to smoking-related cues. Further eye-tracking research is needed to better elucidate these findings.

Among the studies identified for the current synthesis, six studies investigated early attentional processes among people who smoke, and all used direction of initial fixation as the outcome measure of interest (Correa and Brandon, 2016, Creswell and Skrzynski, 2021, Field et al., 2004a, Field et al., 2005, Mogg et al., 2005, Van Rensburg et al., 2009). In each study, people who smoke were shown to direct their gaze toward smoking-related cues more than neutral cues. Many of the studies reviewed used various experimental conditions to examine attentional bias differences among people who smoke. Findings revealed that nicotine deprivation, nicotine dependency, exercise and alcohol consumption are all factors that may have some impact on the strength of fixations to smoking-related cues (Field et al., 2004a, Field et al., 2005, Mogg et al., 2005, Van Rensburg et al., 2009). However, none of the studies found significant differences between experimental conditions (i.e., deprived vs. non-deprived, low dependence vs. moderate dependence, prior alcohol intake vs. no alcohol), suggesting a possible "ceiling effect" in early attentional shifts. In other words, despite differences in state and trait factors, attentional bias to smoking-related cues is a robust finding in people who smoke, and state and trait factors may only impact attentional bias to an extent.

### 4.3 Limitations

This review is not without limitations. Our current review included only 18 studies. With respect to limitations of the studies reviewed, only three studies excluded participants who were on psychotropic medications. Previous studies have shown that psychotropic medication use, such as benzodiazepines, can reduce saccadic velocity and increase latency in eye movements in healthy controls (Reilly et al., 2008). This is due to the sedative effects of many psychotropic drugs on the central nervous system (Reilly et al., 2008). Given the high correlations between nicotine use and psychiatric comorbidities, this is an important methodological consideration for future eye-tracking studies (Farrell et al., 2001). Moreover, only one of the reviewed studies (Creswell and Skrzynski, 2021) conducted a power analysis to determine the sample size needed to detect group differences. Future studies with greater statistical power and larger sample sizes are warranted to yield stronger and more reliable conclusions.

With respect to the limitations of this review, English language and publication bias may have limited the number of available studies included in this review. Secondly, the use of different eye-tracking devices with different spatial and temporal resolutions may have impacted the accuracy of the results. Many studies used eye-tracking devices that required the head to be mounted on a chest rest. While these devices may provide better spatial accuracy, they lack ecological validity (Niehorster et al., 2018). Few studies introduced new, innovative devices to assess eye movements, such as remote or mobile eye-trackers (Baschnagel, 2013, Gamito et al., 2014). These devices allowed participants to move their heads freely. There are several advantages to these types of eye-trackers, such as a more natural assessment of eye gaze; however, there is a risk of data loss when a participant moves their head in non-optimal positions (Niehorster et al., 2018). Nevertheless, there is an imperative need for eye-tracking studies to follow a standardized methodological approach to improve reproducibility, as well as substantiate current findings. This would also allow for accurate comparisons across different substance use disorders. Further, the reviewed studies did not investigate gender/sex differences in attentional bias towards smoking-related cues. Research indicates that women are more likely to smoke due to negative reinforcement than men, which may have implications on attentional bias, particularly across different mood states (Pang and Leventhal, 2013, Weinberger et al., 2010). Understanding these differences could provide valuable insights into gender/sex-specific mechanisms that drive smoking behaviour. We encourage future eye-tracking studies to explore these potential sex/gender differences in attentional bias. Lastly, we were limited to a qualitative review of the data extracted from the selected studies. A meta-analytical synthesis was not deemed appropriate due to the heterogeneity in the outcomes reported. Studies included in this review had variations in stimulus type and presentation times, making comparisons across studies difficult.

## 5 Conclusion

In conclusion, the findings from our current review support previous assertions that smoking-related attentional biases may rely primarily on controlled processes (i.e., maintained attention) more than automatic, preconscious processes (i.e., early attentional shifts) (Field et al., 2004b). These findings have important clinical implications for the treatment of tobacco dependence. Clinicians and other health care providers should be made aware that automatic processes, such as the aberrant allocation of attention toward substance-related cues, may predispose individuals with substance-related disorders towards a lapse or recrurrence of use. Psychological interventions, such as cognitive behavioural therapy, alone may not be sufficient for return to smoking prevention. Instead, clinicians should integrate treatments that assist in reducing maintained attentional bias, such as attentional bias modification, exercise, or non-invasive brain stimulation, to augment current cessation therapies. The findings of this review also underscore the importance of adopting a standardized methodology for future eye-tracking studies to improve the reproducibility and validity of obtained results.

## Funding sources

This research did not receive any specific grant from funding agencies in the public, commercial, or not-for-profit sectors.

## CRediT authorship contribution statement

**Kameron Iturralde:** Writing – review & editing, Writing – original draft. **Laurie Zawertailo:** Writing – review & editing, Supervision, Methodology, Conceptualization. **Noreen Rahmani:** Writing – review & editing, Writing – original draft, Methodology, Investigation, Conceptualization. **Alma Rahimi:** Writing – review & editing, Writing – original draft, Methodology, Investigation, Conceptualization.

## Declaration of Competing Interest

The authors declare that they have no known competing financial interests or personal relationships that could have appeared to influence the work reported in this paper.

## Figures and Tables

**Table:** 

Medline Searches	Embase Searches	PsychInfo Searches
*Nicotine Search Terms*		
"Tobacco Use Disorder"/	Tobacco Dependence/	"Tobacco Use Disorder"/
"Tobacco Use"/	"Tobacco Use"/	
Tobacco Products/	Tobacco/	
Cigar Smoking/	Cigar Smoking/ Or Cigar/	
Cigarette Smoking/	Cigarette Smoking/ Or Cigarette Smoke/ Or Cigarette/	
Electronic Nicotine Delivery Systems/	Electronic Cigarette/	Electronic Cigarettes/
Nicotine Chewing Gum/	Nicotine Gum/ Or Nicotine Lozenge/ Or Nicotine Replacement Therapy/ Or Nicotine Patch/	
Nicotine/	Nicotine/	Nicotine/ Nicotine Withdrawal/
Pipe Smoking/	Pipe Smoking/	
Smokers/		
Smoking Devices/	Smoking Device/	
Smoking Pipes/		
Smoking Water Pipes/	Hookah/	
Smoking/	Smoking/ Or "Smoking And Smoking Related Phenomena"/ Or Adolescent Smoking/ Or Smoking Habit/	
Tobacco Smoking/		Tobacco Smoking/
Tobacco, Smokeless/	Smokeless Tobacco/	Smokeless Tobacco/
Tobacco, Waterpipe/		
Vaping/	Vaping/	
Water Pipe Smoking/	Water Pipe Smoking/	
E-Cigarette Vapor/	Electronic Cigarette Vapor/	
cigarette*.mp.	cigarette*.mp.	cigarette*.mp.
cigar.mp.	cigar.mp.	cigar.mp.
e-cigarette*.mp.	e-cigarette*.mp.	e-cigarette*.mp.
e-cig*.mp.	e-cig*.mp.	e-cig*.mp.
JUUL.mp.	JUUL.mp.	JUUL.mp.
nicotine.mp.	nicotine.mp	nicotine.mp.
smoke*.mp.	smoke*.mp.	smoke*.mp.
smoking.mp.	smoking.mp.	smoking.mp.
tobacco.mp.	tobacco.mp.	tobacco.mp.
TUD.mp.	TUD.mp.	TUD.mp.
vape*.mp.	vape*.mp.	vape*.mp.
vaping.mp.	vaping.mp.	vaping.mp
waterpipe*.mp.	waterpipe*.mp.	waterpipe*.mp.
hookah.mp.	hookah.mp.	hookah.mp.
((e-cigarette or e-cig or nicotine or smok* or tobacco or vape or vaping) adj3 (device* or pipe* or deliver*)).mp.	((e-cigarette or e-cig or nicotine or smok* or tobacco or vape or vaping) adj3 (device* or pipe* or deliver*)).mp.	((e-cigarette or e-cig or nicotine or smoke* or tobacco or vape or vaping) adj3 (device* or pipe* or deliver*)).mp.
((e-cig or e-cigarette or nicotine or tobacco or vape or vaping) adj3 (abuse* or addict* or consume* or consumption or dependen* or disorder* or use*)).mp.	((e-cig or e-cigarette or nicotine or tobacco or vape or vaping) adj3 (abuse* or addict* or consume* or consumption or dependen* or disorder* or use*)).mp.	((e-cig or e-cigarette or nicotine or tobacco or vape or vaping) adj3 (abuse* or addict* or consume* or consumption or dependen* or disorder* or use*)).mp.
*Eye-Movement Search Terms*		
Eye Movements/	Eye Movement/ Or Eye Movement Control/	Eye Movements/ Visual Search/
Fixation, Ocular/	Eye Fixation/ or Ophthalmic Fixation Device/	Eye Fixation/
Pursuit, Smooth/	Smooth Pursuit Eye Movement/	
Saccades/	Saccadic Eye Movement/	
Eye Movement Measurements/	Eye Movement Monitor/ Or Eye Movement Testing Equipment/ Or Functional Vision Testing Equipment/ Or Visual System Examination/	Tracking/
Eye-Tracking Technology/	Eye Tracking/	Visual Tracking/ Visual Displays/ Visual Stimulation/
	Orienting reflex/ or Visual orientation/	Orienting Reflex/
Visual Fields/	Visual Field/ or Peripheral Vision/	Visual Field/ Peripheral Vision/
saccad*.mp.	saccad*.mp.	saccad*.mp.
(smooth adj3 pursuit).mp.	(smooth adj3 pursuit).mp.	(smooth adj3 pursuit).mp.
((eye* or gaze or gazing or ocular or vision or visual) adj3 (display* or fix* or move or moving or movement* or orient* or search* or scan* or system* or stimul* or technology or technologies or test* or time* or timing or reflex* or track*)).mp.	((eye* or gaze or gazing or ocular or vision or visual) adj3 (display* or fix* or move or moving or movement* or orient* or search* or scan* or system* or stimul* or technology or technologies or test* or time* or timing or reflex* or track*)).mp.	((eye* or gaze or gazing or ocular or vision or visual) adj3 (display* or fix* or move or moving or movement* or orient* or search* or scan* or system* or stimul* or technology or technologies or test* or time* or timing or reflex* or track*)).mp.
(dwell* adj3 (time* or timing)).mp.	(dwell* adj3 (time* or timing)).mp.	(dwell* adj3 (time* or timing)).mp.
*Attentional Bias Search Terms*		
Association Learning/	Associative Learning/	Free Association/ Associative Processes/
Attention/	Selective Attention/ Attention Test/ Attention Network/ Visual Attention/	Attention/ Attentional Capture/ Divided Attention/ Focused Attention/ Selective Attention/ Attention Span/ Visual Attention/
Attentional Bias/	Attentional Bias/	Attentional Bias/
	Cognitive Bias/	Cognitive Discrimination/ Cognitive Bias/
Cues/		Cues/ Pictorial Stimuli/
Reaction Time/	Reaction Time/	Reaction Time/
Signal Detection, Psychological/	Signal Detection/	"Signal Detection (Perception)"
Stroop Test/	Stroop Test/	Stroop Color Word Test/ Stroop Effect/
Visual Perception/	Visual Discrimination/	orientingg Visual Discrimination/
attention*.mp.	attention*.mp.	attention*.mp.
cue*.mp.	cue*.mp.	cue*.mp.
bias*.mp.	bias*.mp.	bias*.mp.
dot probe.mp.	dot probe.mp.	dot probe.mp.
incentive salience.mp.	incentive salience.mp.	incentive salience.mp.
reaction* time*.mp.	reaction* time*.mp.	reaction* time*.mp.
response bias.mp.	response bias.mp.	response bias.mp.
stroop.mp.	stroop.mp.	stroop.mp.
visual probe.mp.	visual probe.mp.	visual probe.mp.
visual discriminat*.mp.	visual discriminat*.mp.	visual discriminat*.mp.
pictorial stimul*.mp.	pictorial stimul*.mp.	pictorial stimul*.mp.
((attention or cognit* or ocular or response or reaction or visual or vision) adj3 (bias* or cognit* or latency or latencies or preference* or process* or select* or time*)).mp.	((attention or cognit* or ocular or response or reaction or visual or vision) adj3 (bias* or cognit* or latency or latencies or preference* or process* or select* or time*)).mp.	((attention or cognit* or ocular or response or reaction or visual or vision) adj3 (bias* or cognit* or latency or latencies or preference* or process* or select* or time*)).mp.
((select* or maintain* or visual) adj3 attention).mp.	((select* or maintain* or visual) adj3 attention).mp.	((select* or maintain* or visual) adj3 attention).mp.
((initial or maintain*) adj3 orientation).mp.	((initial or maintain*) adj3 orientation).mp.	((initial or maintain*) adj3 orientation).mp.
(signal adj3 detection*).mp.	(signal adj3 detection*).mp.	(signal adj3 detection*).mp.
